# Long‐Term Effects of High‐Intensity Aerobic Training on Metabolic Syndrome: An 8‐Year Follow‐Up Randomized Clinical Trial

**DOI:** 10.1002/jcsm.13780

**Published:** 2025-04-02

**Authors:** Felix Morales‐Palomo, Alfonso Moreno‐Cabañas, Laura Alvarez‐Jimenez, Diego Mora‐Gonzalez, Ricardo Mora‐Rodriguez

**Affiliations:** ^1^ Exercise Physiology Lab at Toledo, Faculty of Sport Sciences University of Castilla–La Mancha Toledo Spain; ^2^ Centre for Nutrition, Exercise, and Metabolism University of Bath Bath UK; ^3^ Department of Anesthesiology University of California San Diego California USA; ^4^ Department of Nursing, Physiotherapy and Occupational Therapy University of Castilla–La Mancha Toledo Spain

**Keywords:** ageing, drug utilization, exercise intervention, metabolic syndrome, physical fitness

## Abstract

**Background:**

Metabolic syndrome (MetS) is a set of five cardiometabolic risk factors that typically worsen with age. One exercise‐training programme is effective at improving those factors in middle‐aged individuals with MetS. To our knowledge, exercise‐training efficacy as MetS individuals age has not been explored. This study determined the effectiveness of a periodized exercise training programme for individuals with MetS after a follow‐up period of 8 years.

**Methods:**

Forty‐seven individuals with MetS were block‐randomized into an EXERCISE (*n* = 22, 52 ± 8 years old, 23% women) or a CONTROL group (*n* = 25, 53 ± 8 years old, 32% women). Both groups received standard health care, including medical counselling and lifestyle advice at least every 6 months, while participants in EXERCISE also underwent a supervised exercise programme. The intervention lasted 8 years and consisted of 4 months per year (November to March) of high‐intensity interval training thrice weekly. At baseline, and after 4 and 8 years of treatment, we assessed body composition, MetS components (i.e., MetS *Z* score), medication use, cardiorespiratory fitness (CRF; assessed by VO_2MAX_) and maximal leg cycling power output (W_MAX_).

**Results:**

Paradoxically, MetS *Z* score and body weight were reduced after 8 years (subjects aged from 52 to 60 years old) in both groups (time effect *p* < 0.001 and *p* = 0.008; time × group interaction *p* = 0.253 and *p* = 0.130). However, in those 8 years, the medicine use score increased threefold in CONTROL (137% increase; from 1.7 to 3.9; *p* < 0.001) while it did not change in EXERCISE (33%; from 2.0 to 2.7; *p* = 0.066). In 8 years, CRF and W_MAX_ increased in EXERCISE by 14% (3.4 ± 5.6 mL·kg^−1^·min^−1^) and 4% (7 ± 37 W) while decreasing in CONTROL by −7% (−1.6 ± 3.4 mL·kg^−1^·min^−1^) and −14% (−24 ± 27 W) being different between groups after 4 and 8 years (both time × group interaction *p* = 0.002). Pearson correlations showed that MetS *Z* score improvements were significantly associated with increases in medication use score in the CONTROL group (*r* = 0.491; *p* = 0.013) and with W_MAX_ enhancement in the EXERCISE group (*r* = 0.613; *p* = 0.002).

**Conclusions:**

Our data suggest that annual exercise training has similar clinical efficacy to triple oral medication for the management of MetS in individuals aged 50 to 60 years. The health of individuals with cardiometabolic disorders can be maintained as they age by increasing medication or by participating in an annual intensive exercise programme.

## 1 Introduction

Metabolic syndrome (MetS) is a complex of interconnected cardiometabolic disorders that typically increase with age [1]. Not only does the prevalence of MetS increase with age [2] but having MetS at any given age results in elevation in DNA markers of premature epigenetic ageing [3]. Thus, it is likely that MetS accelerates the deterioration of the cardiometabolic system beyond the natural decline in function associated with ageing. A hypocaloric diet, exercise and oral medication therapy are the foundations for MetS clinical management [4]. It is unclear if those combined therapies are similarly effective as individuals age. MetS is a great model for a comprehensive study of the impact of ageing, exercise and medication because it has defined thresholds for clinical risk factors such as arterial pressure, blood glucose, triglycerides, HDL and central obesity [5].

Compounding the effects of ageing, the medication that ageing people habitually take, could interfere with training adaptations. It is well established that some of the prescriptions that middle‐aged and old individuals regularly take to control atherosclerotic cardiovascular risk, blunt the positive adaptation of training in muscle mitochondria. For instance, statins [S41] and metformin [S42] lessen exercise‐induced mitochondrial adaptation and whole‐body increases in cardiorespiratory fitness (CRF, as assessed by VO_2MAX_) after high‐intensity aerobic training [6, 7]. Because mitochondrial capacity correlates with insulin sensitivity [8], the combination of ageing and certain oral medications may prevent exercise training from alleviating insulin resistance [9]. Some studies suggest that metformin blunts insulin‐like growth factor 1 (IGF‐1) [7] and muscle strength gains following training in middle‐aged and older individuals [10], which would result in reduced protection against ageing. In contrast, oral medicines that lower blood pressure could allow more intense training potentiating training adaptations [11]. Thus, medication is a factor that should always be factored in when reporting the health‐promoting effects of exercise training in the ageing population.

Regular exercise promotes healthspan in the ageing population [S43] and could even delay mortality [12]. Training programme characteristics influence the magnitude of skeletal muscle and cardiometabolic adaptations to exercise in a dose–response manner [13]. Strong evidence exists that aerobic exercise intensity mediates mitochondrial adaptations to exercise and improvements in maximal aerobic capacity (VO_2MAX_ [14]). Additionally, repeated exposure to an exercise dose is required to consolidate transient improvements in health parameters into durable health adaptations in MetS patients [15]. The maintenance of both, CRF and muscular strength while ageing are probably the most critical health‐related outcome measures of healthspan and are strongly associated with reduced mortality due to cardiovascular diseases [16]. Previous studies have found a strong association between low levels of CRF and muscular strength and increased incidence of MetS in men and women [17, 18]. Conversely, higher levels of CRF [19] and muscular strength [20] protect against the development of MetS as individuals age.

Several studies endorse that a 4‐ to 6‐month intense aerobic exercise training programme effectively reduces MetS components [S44, S45]. However, those studies do not integrate in their findings the concurrent effects of oral medication or the effects of ageing in these individuals. Other rigorously controlled randomized trials in obese participants (Diabetes Prevention Program [DPP] [21], Look AHEAD [22], U‐TURN [23] and PROPEL [24]) show a reduction in cardiometabolic risk factors and medication use when combining physical activity and diet to achieve clinically relevant body weight loss (i.e., > 5%). However, these interventions require a drastic lifestyle change rarely sustained after the experiment is completed [25]. In contrast, lifestyle interventions based on incorporating supervised exercise programmes could correct several risk factors simultaneously and be sustainable in time.

We recently reported that yearly repetition of a 4‐month aerobic training programme for five consecutive years improves MetS without requiring large body weight loss [26]. However, the time frame of our previous study was too short to assess the effects of ageing on exercise‐related health outcomes. In the present study, we followed a sample of individuals with MetS as they aged for 8 years (52–60 years old). We block‐randomized the sample into a control group under lifestyle medical counselling and a group that yearly completed 4 months of supervised high‐intensity interval aerobic training. This RCT design allowed us to assess whether the decline in physical fitness (cardiovascular and muscular) was due to the effects of ageing per se or to a lack of supervised exercise training. Our main hypothesis was that short‐term ageing (from 50–60 years old) would significantly worsen MetS factors, but exercise training could compensate for the ageing‐related deterioration of the cardiometabolic systems.

## 2 Methods

### 2.1 Participants

Forty‐seven middle‐aged (53 ± 8 years) overweight and obese subjects (body mass index [BMI], 33.4 ± 4.2 kg·m^−2^) with MetS criteria completed the study. Participants were previously inactive (< 150 min·week^−1^ of the moderate‐intensity activity assessed by 7‐d IPAQ [S46]. MetS was defined according to the updated International Diabetes Federation 2009 criteria using population Europid waist circumference cutpoints [27]. Exclusion criteria were untreated cardiovascular or renal disease or any condition associated with exercise intolerance. The protocol did not require any method changes during the follow‐up period. All subjects provided written, witnessed and informed consent under a protocol approved by the local Virgen de la Salud Hospital's Ethics Committee and according to the Declaration of Helsinki. This is a substudy part of a larger clinical trial evaluating the effects of interactions of medicine and exercise in individuals with MetS (ClinicalTrials.gov Identifier: NCT03019796). The main study outcomes were MetS *Z* score and medication use score. We maintained the targeted outcomes during the experiment.

### 2.2 Experimental Design

**FIGURE 1:**
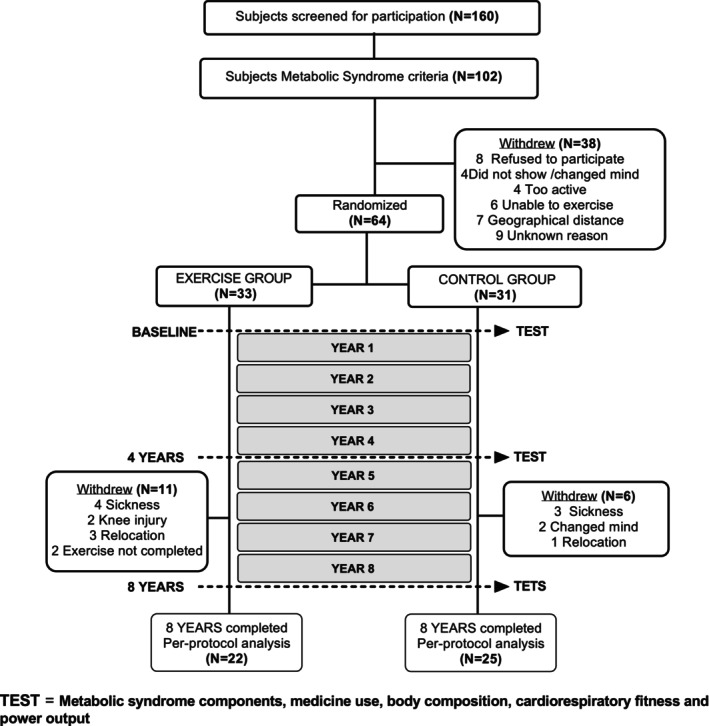
CONSORT (Consolidated Standards of Reporting Trials) schematic representation of the study procedures.

Following a parallel group randomized controlled trial design, participants were recruited, clinically screened and tested in compliance with CONSORT (i.e., Consolidated Standards of Reporting Trials statement; Figure 1). After baseline assessment, 64 participants were stratified in blocks using a specific randomization software. An independent statistician determined a computer‐generated random number sequence. The sequence was given to a research team member who was not involved in the study procedures. Randomization was stratified by age (< 50 and ≥ 50 years), number of MetS factors (there, four and five Mets components) and BMI (< 30 kg/m^2^ and ≥ 30 kg/m^2^). Within each block, participants were randomly assigned to the exercise training (EXERCISE) or standard medical care (CONTROL) groups in a 1:1 ratio. Physiological, anthropometric and blood biochemistry measurements were taken on three occasions: before exercise intervention (baseline) and after 4 and 8 years of treatment. Additional interim analysis was performed after 5 years of follow‐up, which provided information of the short‐term effects of yearly exercise training on cardio‐metabolic health [26, 28]. Body composition and physiological measurements were performed at the Exercise Physiology Laboratory facilities, and blood biochemical variables were assessed at Eurofins Megalab laboratory (Toledo, Spain). Pre‐training data were always collected in early November to avoid seasonal effects. All participants received attention from the Spanish healthcare system, including medical counselling and lifestyle advice at least every 6 months. Physicians were not included in the research group and were naïve to the experimental group assignment. Conversely, personnel responsible for measurements and intervention administration were not blinded to the group assignment (open‐label).

### 2.3 Intervention

Both groups received standard health care while, in addition, participants in EXERCISE underwent a supervised exercise programme (3 sessions·per week) at the sports facilities of the University. Training consisted of high‐intensity interval training (HIIT) on stationary bikes for 4 months yearly (mid‐November to mid‐March) for eight consecutive years. Each exercise session included a 10‐min warm‐up at 70% of peak heart rate (HR_MAX_) followed by 4 × 4‐min intervals at 90% of HR_MAX_ interspersed with a 3‐min active recovery at 70% of HR_MAX_ and a 5‐min cool‐down period. Heart rate (HR) was continuously displayed on a large screen (Seego Realtrack Sytems, Almería, Spain) and participants self‐adjusted the workload to reach their HR target. A research team member monitored each session to ensure adherence to the training scheme. At each evaluation window (baseline, Year 4 and Year 8), before starting the exercise intervention period, participants completed a 3‐day food diary (CESNICD v1.0; Barcelona, Spain) and physical activity pattern (Polar Electro, Kempele, Finland) for 1 week (i.e., the daily number of steps, standing time and supine resting time; see Table S1).

### 2.4 MetS Components and Body Composition

Patients arrived at the laboratory in the morning after an overnight fast. For EXERCISE, post‐training measurements were scheduled at least 48 h after the last exercise training session. Nude body weight (Hawk; Metler, Toledo, OH, USA), height (Stadiometer; Secca 217, Hamburg, Germany) and waist circumference (flexible tape) were measured. Fat mass (FM) and fat‐free mass (FFM) were determined by bioelectrical impedance analysis (Tanita bc‐418; Tanita Corp, Tokyo, Japan). After 10 min of supine rest, blood pressure was measured in triplicate using a calibrated ECG‐gated electro‐sphygmomanometer (Tango, Suntec Medical; NC). Following, a 7‐mL blood sample was collected to determine serum glucose, insulin and lipid levels (triglycerides, total cholesterol, HDL and LDL‐cholesterol). Insulin sensitivity was calculated using the homeostasis model assessment for insulin resistance (HOMA‐IR). Sex‐specific *Z* scores were calculated for each MetS criterion using the group SD, with the sum of the *Z* scores for each MetS component divided by six to compile the MetS risk score with units of SD. The equations used to calculate the MetS *Z* score were:

Men's MetS *Z* score = [(40 − HDL‐cholesterol)/SD] + [(triglycerides − 150)/SD] + [(glucose − 100)/SD] + [(waist circumference − 94)/SD] + [(systolic blood pressure − 130)/SD] + [(diastolic blood pressure − 85)/SD].

Women's MetS *Z* score = [(50 − HDL‐cholesterol)/SD] + [(triglycerides − 150)/SD] + [(glucose − 100)/SD] + [(waist circumference − 80)/SD] + [(systolic blood pressure − 130)/SD] + [(diastolic blood pressure − 85)/SD].

### 2.5 Cardiorespiratory Fitness and Maximal Power Output

Maximal oxygen uptake (VO_2MAX_), maximal cycling power (W_MAX_) and maximal HR (HR_MAX_) were assessed during a graded exercise test (GXT) on an electronically braked cycle ergometer (Ergoselect 200; Ergoline, Germany) using indirect calorimetry (Quark b^2^; Cosmed, Italy). Heart electrical activity was continuously monitored using a standard 12‐lead ECG (Quark T12; Cosmed). After 3 min of warm‐up at 30 W for women and 50 W for men, the workload increased every minute (15 W for women and 20 W for men) until volitional exhaustion. This test was followed by a verification test at 110% of the maximal workload reached to ensure the achievement of VO_2MAX_ [29].

### 2.6 Medication Use

Participants were supervised by their primary care physician following the Spanish Society of Family and Community Medicine guidelines for MetS treatment [S47]. These guidelines include lifestyle advice, blood analysis every 6 months and pharmacological prescriptions adjusted to blood chemistry, blood pressure values and body weight evolution. Physicians were not included in the research group and were naïve to group allocation of the experiment. Participants brought all prescription medication to the laboratory at the three data collection visits to ensure recording accuracy. Only medicines to control MeS factors were registered. We used the defined daily dose (DDD; [S48]) by the international standard in drug utilization research (DUR) as recommended by the World Health Organization (WHO) since 1996. The DDD is the recommended daily dose for that specific drug in adults based on the main active chemical independent of manufacturer presentation. The use of DDD enabled us to monitor medication evolution in patients taking different brands of the same drug.

We developed a medicine use score that integrates the number and dose of medications taken by each participant, as follows [26]:

$$\begin{array}{c} \text{Medicine}\;\mathrm{use}\;\text{score}=\left[\text{medicine}\;1\;\left(\text{subject used dose}/\mathrm{DDD}\;\text{index}\right)\right]\\ \;+\left[\text{medicine}\;2\;\left(\text{subject used dose}/\mathrm{DDD}\;\text{index}\right)\right]\\ \;+\ldots \;\ldots \;\ldots \\ \;+\left[\text{medicine}\;n\;\left(\text{subject used dose}/\mathrm{DDD}\;\text{index}\right)\right]. \end{array}$$

### 2.7 Statistical Analysis

We used per‐protocol analysis, and only patients who completed the treatment protocol were included in the statistical analysis. Sample size calculation was based on MetS *Z* score data changes in individuals with MetS who completed a 16‐week exercise training programme for three consecutive years [15]. Assuming 80% power and an α‐error probability of 0.05, it was calculated that 16 patients would be required to detect a significant effect of long‐term exercise training on improvement in the MetS *Z* score. Because the present study had a longer follow‐up period, we doubled the initially randomized sample size. Data are presented as mean ± SD. Kolmogorov–Smirnov test revealed that all variables showed a normal distribution except the number of prescribed medications and medication use score. Mixed‐design ANOVA without multiple imputations was used to analyse differences across time (repeated measures) and between experimental groups (CONTROL vs. EXERCISE) in anthropometric, MetS factors, CRF and muscular power levels. Despite our previous observation that the metabolic and cardiovascular responses to 4 months of HIIT in MetS patients are similar in men and women [S49], an analysis of covariance (ANCOVA) was performed to eliminate the potential influence of sex on the primary outcomes. When the time‐by‐group interaction was significant, vertical multiple comparisons were performed using Bonferroni post‐hoc testing. The 95% confidence intervals (CI) were also calculated. To improve the interpretation of the differences, the effect size of time and time × group interaction were calculated using eta squared (*η* ^2^). The effect size obtained from *η* ^2^ was considered large if ≥ 0.14, moderate ≥ 0.06 and small if < 0.06. The difference within and between groups in medication use (per type of drug) at 4‐ and 8‐year follow‐up compared with baseline was tested using a Cochran's *Q* test and chi‐square (*χ* ^2^), respectively. For nonnormally distributed quantitative differences, the median changes of the medication use score from baseline to 4‐ and 8‐year follow‐up were tested using a Friedman (within‐group) test and Mann–Whitney *U* test (between‐group). Pearson correlation coefficients (*r*) were used to test the association among variables. Stepwise linear regression analysis was used to examine the strength of the associations between the MetS *Z* score and the other variables. Statistical analyses were performed using SPSS (IBM Corporation, Armonk, New York, USA), version 28, and the statistical significance level was set at *α* < 0.05.

## 3 Results

### 3.1 Subjects and Exercise Characteristics

A flow diagram of study participants is shown in Figure 1. All participants were white living in southern Europe and women participants comprised 27% of all subjects. Data were analysed without sex differentiation since all women were postmenopausal and were not taking hormonal replacement therapy, and their responses did not differ from men's responses in the main outcomes of the study (time × sex interaction; MetS *Z* score, *p* = 0.53; medication use score, *p* = 0.30; body weight [kg], *p* = 0.66; VO_2MAX_ [L·min^−1^], *p* = 0.56; and W_MAX_ [W], *p* = 0.66). During the 8 years of follow‐up, participants in EXERCISE attended at least 90% of the prescribed exercise sessions, and no exercise‐related adverse effects were noted. Subjects declared no involvement in a regular exercise programme during the months without supervised exercise (8 months per year). There were no significant differences in calorie intake or physical activity levels between the groups at any time (all *p* > 0.05; see Table S1).

### 3.2 MetS Components and Other Health Parameters

**FIGURE 2:**
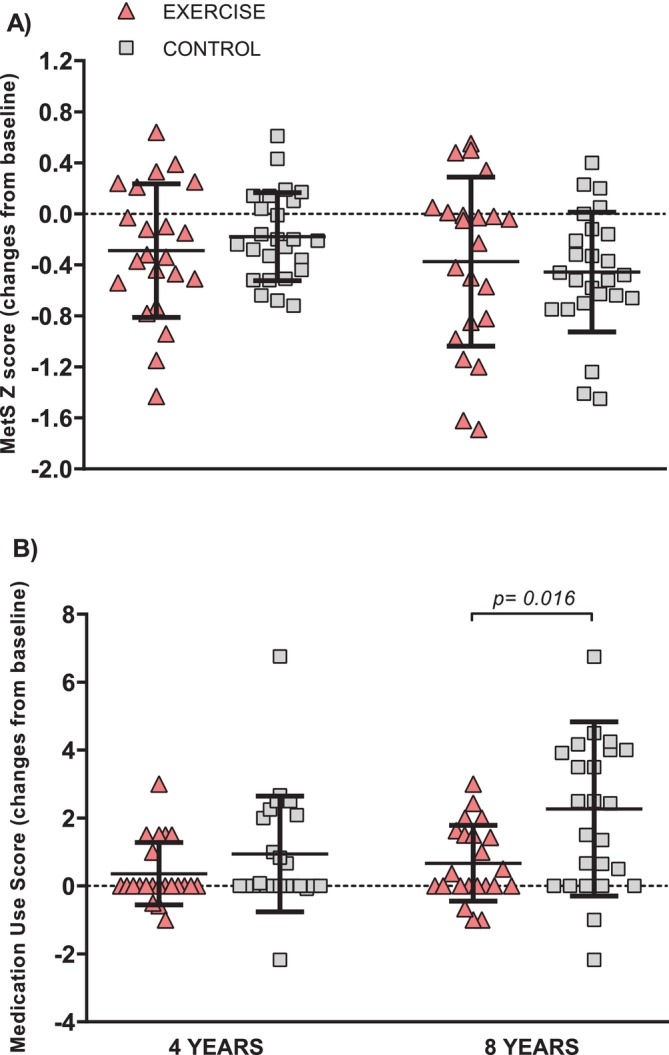
Changes in MetS *Z* score (A) and medication use score (B) after 4 and 8 years in the EXERCISE and CONTROL. Values are presented as dot plots with mean ± SD.

The evolution of MetS components and related parameters during 8 years of follow‐up are depicted in Table 1. A significant improvement with time was observed for HDL‐cholesterol (*p* = 0.009), systolic blood pressure (*p* = 0.001) and diastolic blood pressure (*p* < 0.001), when both groups were pooled together. However, only a significant time × group interaction effect was found in waist circumference (*p* = 0.049) with a tendency to increase after 8 years in the CONTROL group (3%; 95% CI −0.02 to 6.11 cm; *p* = 0.053). MetS *Z* score was similarly reduced over time in both groups (*p* < 0.001) and changes from baseline between the EXERCISE and CONTROL groups were not statistically different (Figure 2A).

**TABLE 1:** Eight‐year evolution of anthropometric, metabolic syndrome factors and other clinical variables by group. Data are presented as mean ± SD.

	EXERCISE (*n* = 22)			CONTROL (*n* = 25)			*p* (*η* ^2^)	
	Baseline	4 years	8 years	Baseline	4 years	8 years	Time	Time × group
BMI (kg·m^−2^)	33.4 ± 4.6	32.9 ± 4.4	31.9 ± 3.8	33.5 ± 3.9	33.7 ± 4.3	33.4 ± 4.2	**0.047 (0.13)**	0.136 (0.09)
Weight (kg)	95.6 ± 14.1	93.8 ± 12.7	90.3 ± 11.6	92.1 ± 15.3	92.6 ± 16.1	90.9 ± 3.2	**0.008 (0.20)**	0.130 (0.09)
Fat mass (kg)	32.6 ± 8.3	31.3 ± 8.0	29.5 ± 7.9	33.2 ± 7.3	33.2 ± 7.5	32.7 ± 7.6	0.103 (0.10)	0.317 (0.05)
Trunk fat (kg)	19.1 ± 4.8	18.2 ± 4.2	17.4 ± 4.3	19.8 ± 4.6	19.3 ± 4.5	20.0 ± 5.0	0.169 (0.08)	0.079 (0.11)
Fat‐free mass (kg)	62.9 ± 9.9	62.5 ± 9.6	60.8 ± 10.0	58.9 ± 11.5	59.5 ± 11.7	58.2 ± 11.5	**0.035 (0.14)**	0.308 (0.05)
Fat‐free mass right leg (kg)	10.3 ± 1.8	10.7 ± 1.8	10.5 ± 1.9	9.3 ± 2.3	9.3 ± 2.3	9.1 ± 2.1	0.074 (0.11)	0.112 (0.10)
Waist circumference (cm)	109.2 ± 9.3	109.6 ± 9.0	107.9 ± 8.7	108.9 ± 9.9	110.8 ± 11.8	112.1 ± 2.2	0.425 (0.04)	**0.049 (0.13)**
Glucose (mg·dL^−1^)	118.4 ± 32.9	120.5 ± 48.5	115.1 ± 32.1	112.0 ± 21.0	118.1 ± 36.1	115.5 ± 49.8	0.561 (0.03)	0.768 (0.01)
Triglycerides (mg·dL^−1^)	140.8 ± 64.6	131.9 ± 45.2	144.3 ± 66.0	131.2 ± 85.3	142.7 ± 90.0	108.5 ± 44.9	0.520 (0.03)	0.053 (0.13)
HDL‐c (mg·dL^−1^)	42.5 ± 11.1	47.0 ± 11.0	47.3 ± 10.2	43.2 ± 16.4	47.2 ± 17.2	45.4 ± 12.5	**0.009 (0.19)**	0.725 (0.02)
Systolic blood pressure (mmHg)	133.1 ± 16.9	127.8 ± 14.9	125.0 ± 12.7	134.4 ± 15.1	129.3 ± 14.1	123.2 ± 13.6	**0.001 (0.26)**	0.648 (0.02)
Diastolic blood pressure (mmHg)	81.0 ± 10.2	75.5 ± 8.7	72.9 ± 7.9	85.2 ± 11.4	81.5 ± 7.8	77.4 ± 8.3	**< 0.001 (0.39)**	0.736 (0.01)
MetS *Z* score (SD)	0.36 ± 0.60	0.07 ± 0.65	‐0.01 ± 0.65	0.44 ± 0.49	0.26 ± 0.51	−0.02 ± 0.54	**< 0.001 (0.36)**	0.253 (0.06)
Insulin (μIU·mL^−1^)	13.4 ± 4.9	13.3 ± 6.8	10.7 ± 4.4	12.1 ± 4.3	12.9 ± 6.5	11.4 ± 4.9	**0.003 (0.23)**	0.259 (0.06)
HOMA‐IR	4.0 ± 2.0	4.1 ± 2.6	3.1 ± 1.7	3.4 ± 1.3	3.7 ± 2.0	3.2 ± 1.6	**0.011 (0.19)**	0.228 (0.07)
T‐Cholesterol (mg·dL^−1^)	191.6 ± 36.0	180.1 ± 35.4	180.0 ± 36.3	198.2 ± 43.2	190.2 ± 35.3	178.4 ± 43.7	**0.049 (0.13)**	0.435 (0.04)
LDL‐c (mg·dL^−1^)	120.9 ± 30.1	106.8 ± 31.8	103.8 ± 35.0	128.7 ± 31.6	114.4 ± 27.8	111.2 ± 37.0	**0.002 (0.24)**	0.999 (0.00)
VO_2MAX_ (L·min^−1^)	2.32 ± 0.66	2.52 ± 0.64^a^	2.50 ± 0.66	2.09 ± 0.53	2.09 ± 0.58^c^	1.94 ± 0.65^a^, ^b^, ^c^	**0.029 (0.15)**	**0.011 (0.18)**
VO_2MAX_ (mL·kg·min^−1^)	24.4 ± 6.9	27.1 ± 7.4^a^	27.8 ± 7.1^a^	22.7 ± 4.3	22.5 ± 4.5^c^	21.1 ± 5.5^c^	0.101 (0.10)	**0.002 (0.24)**
Heart rate max (beat min^−1^)	156 ± 15	163 ± 15^a^	155 ± 14^b^	153 ± 16	150 ± 18^c^	147 ± 15^a^	**0.001 (0.27)**	**0.020 (0.16)**
Maximal power output (W)	185 ± 57	202 ± 56^a^	191 ± 54	170 ± 50	163 ± 51^c^	146 ± 51^a^, ^b^, ^c^	**< 0.001 (0.35)**	**0.002 (0.24)**
Maximal power output (W·kg)	1.9 ± 0.6	2.2 ± 0.7^a^	2.1 ± 0.6	1.8 ± 0.4	1.8 ± 0.4^c^	1.6 ± 0.4^a^, ^b^, ^c^	**0.017 (0.17)**	**0.001 (0.28)**

^a^ Significant change from baseline within each group.

^b^ Significant change from 4 years within each group.

^c^ Significant difference between EXERCISE and CONTROL groups at that time point (all *p* < 0.05).

### 3.3 Medication Use

The changes in pharmacological treatment during the 8‐year follow‐up are depicted in Table 2. The percentage of subjects in the CONTROL group under blood pressure‐lowering (*p* = 0.011), lipid‐lowering (*p* = 0.022), glucose‐lowering (*p* = 0.022) and total medication (p = 0.011) increased from baseline after 8 years. The total number of prescribed medications increased in the CONTROL group from 4 to 8 years (*p* = 0.001). At 8 years of follow‐up, the number of lipid‐lowering (*p* = 0.036) and total medication (*p* = 0.033) were higher in CONTROL than in the EXERCISE group. The medicine use score of antihypertensive, lipid‐lowering and total medicine increased above baseline after 8 years in the CONTROL (1.08; 91% increase; *p* = 0.016, 0.72; 233% increase; *p* = 0.006, and 2.27; 137% increase; *p* < 0.001, respectively). Changes from baseline in medicine use scores were statistically larger in CONTROL than EXERCISE groups after 4 and 8 years for lipid‐lowering (*p* = 0.026 and *p* = 0.023) and total medication (*p* = 0.016, Figure 2B).

**TABLE 2:** Eight‐year evolution of medication use by group. Data are presented as number of subjects taking that drug (%) and the median of medication use (IQR; interquartile range).

	EXERCISE (*n* = 22)			CONTROL (*n* = 25)		
	Baseline	4 years	8 years	Baseline	4 years	8 years
Total medications						
Subjects under pharmacological treatment, *n* (%)^a^, ^b^	16 (73)	17 (77)	17 (77)	12 (48)	15 (60)	18 (72)^e^
Number of prescribed medications, median (IQR)^c^, ^d^	1.7 (1.3)	2.0 (1.3)	2.4 (3.0)	1.5 (2.5)	2.1 (2.0)	3.2 (3.0)^e^, ^f^, ^g^
Medicine use score, median (IQR)^c^, ^d^	2.0 (1.9)	2.4 (1.7)	2.7 (3.4)	1.7 (2.4)	2.6 (2.6)	3.9 (3.4)^e^, ^f^, ^g^
Lipid‐lowering						
Subjects under pharmacological treatment, *n* (%)^a^, ^b^	10 (45)	9 (41)	9 (41)	12 (48)	15 (60)	17 (68)^e^
Number of prescribed medications, median (IQR)^c^, ^d^	0.5 (1.0)	0.4 (1.0)	0.4 (1.0)	0.5 (1.0)	0.6 (1.0)	0.9 (1.0)^g^
Medicine use score, median (IQR)^c^, ^d^	0.4 (0.8)	0.4 (0.7)	0.5 (0.8)	0.3 (0.7)	0.6 (1.0)f	1.0 (1.9)^e^, ^g^
Glucose‐lowering						
Subjects under pharmacological treatment, *n* (%)^a^, ^b^	4 (18)	6 (27)	7 (32)	3 (12)	6 (24)	8 (32)^e^
Number of prescribed medications, median (IQR)^c^, ^d^	0.2 (0.0)	0.4 (1.0)	0.5 (1.0)	0.3 (0.0)	0.5 (0.5)	0.7 (1.5)
Medicine use score, median (IQR)^c^, ^d^	0.1 (0.0)	0.3 (0.4)	0.3 (0.4)	0.2 (0.0)	0.3 (0.2)	0.6 (0.6)
Blood pressure‐lowering						
Subjects under pharmacological treatment, *n* (%)^a^, ^b^	16 (73)	17 (77)	17 (77)	12 (48)	15 (60)	18 (72)^e^
Number of prescribed medications, median (IQR)^c^, ^d^	1.0 (1.3)	1.2 (1.3)	1.4 (1.3)	0.8 (1.0)	1.0 (2.0)	1.6 (3.0)^e^
Medicine use score, median (IQR)^c^, ^d^	1.5 (2.0)	1.6 (2.0)	1.9 (2.6)	1.2 (2.0)	1.7 (2.3)	2.3 (3.8)^e^

^a^ Between‐group difference using *χ* ^2^ test.

^b^ Within‐group difference using Cochran's *Q* test.

^c^ Between‐group difference using Mann–Whitney *U* test.

^d^ Within‐group difference using Friedman test.

^e^ Significant change from baseline within each group.

^f^ Significant change from 4 years within each group.

^g^ Significant difference between EXERCISE and CONTROL groups at that time point (all *p* < 0.05).

### 3.4 Body Weight and Composition

The evolution in body weight and body composition in each group are depicted in Table 1. There was a significant time effect in BMI (*p* = 0.047), weight (*p* = 0.008) and fat‐free mass (*p* = 0.035); however, there were no significant time‐by‐group interaction effects (*p* = 0.136, *p* = 0.130 and *p* = 0.308, respectively).

### 3.5 Cardiorespiratory Fitness and Maximal Power

**FIGURE 3:**
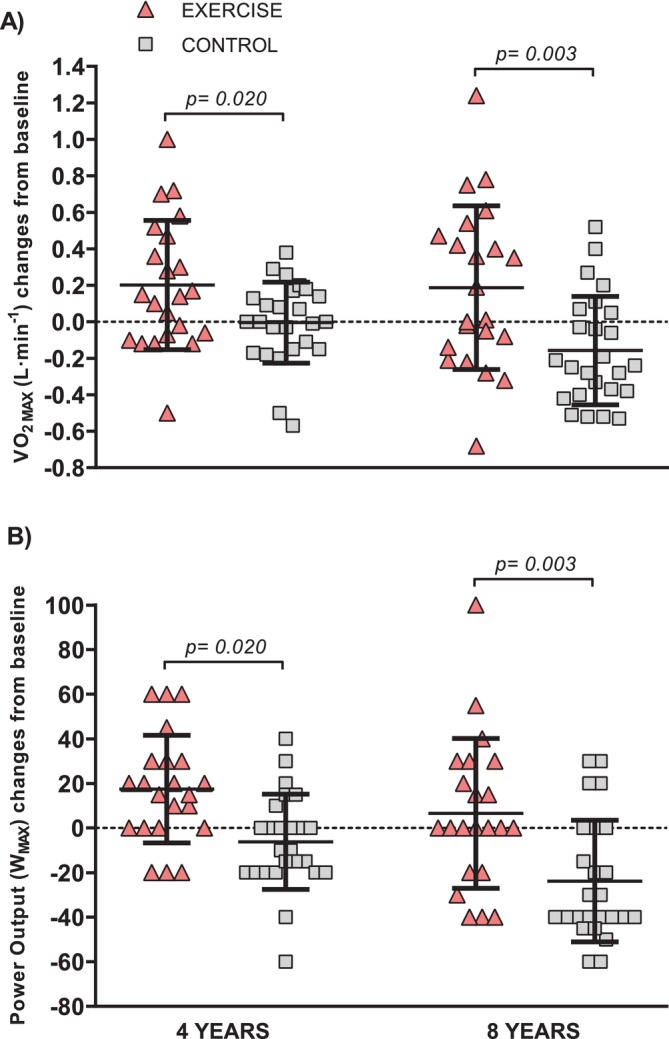
Changes in VO_2MAX_ (A) and W_MAX_ (B) after 4 and 8 years in the EXERCISE and CONTROL. Values are presented as dot plots with mean ± SD.

CRF (i.e., VO_2MAX_), maximal power output (W_MAX_) and HR max (HR_MAX_) evolution during 8 years of follow‐up are shown in Table 1. A significant time × group interaction effect emerged for VO_2MAX_ (*p* = 0.011), W_MAX_ (*p* = 0.002) and HR_MAX_ (*p* = 0.020). The EXERCISE group improved their VO_2MAX_ after 4 years (9%; 95% CI 0.05 to 0.36 L·min^−1^; *p* = 0.006) and after 8 years (8%; 95% CI −0.01 to 0.39 L·min^−1^; *p* = 0.069). Contrarily, the CONTROL group had a significant reduction in VO_2MAX_ after 8 years (−7%; 95% CI −0.34 to −0.02 L·min^−1^; *p* = 0.042) and from 4 to 8 years (−8%; 95% CI −0.30 to −0.01 L·min^−1^; *p* = 0.038). W_MAX_ increased in EXERCISE after 4 years (9%; 95% CI 5 to 30 W, p = 0.002) while decreasing in the CONTROL group after 8 years (−14%; 95% CI −39 to −9 W; *p* = 0.001) and from 4 to 8 years (−11%; 95% CI −28 to −7 W; *p* < 0.001). Changes from baseline in VO_2MAX_ and W_MAX_ between the EXERCISE and CONTROL groups were statistically different at 4 years (*p* = 0.020) and 8 years (*p* = 0.009; Figure 3A,B). The CONTROL group reduced their HR_MAX_ after 8 years (4%; 95% CI −10 to −1 beats min^−1^; *p* = 0.021) while the EXERCISE group maintained HR_MAX_ resulting in a significant time by group interaction (*p* = 0.011).

### 3.6 Correlations and Multiple Regression Analysis

**FIGURE 4:**
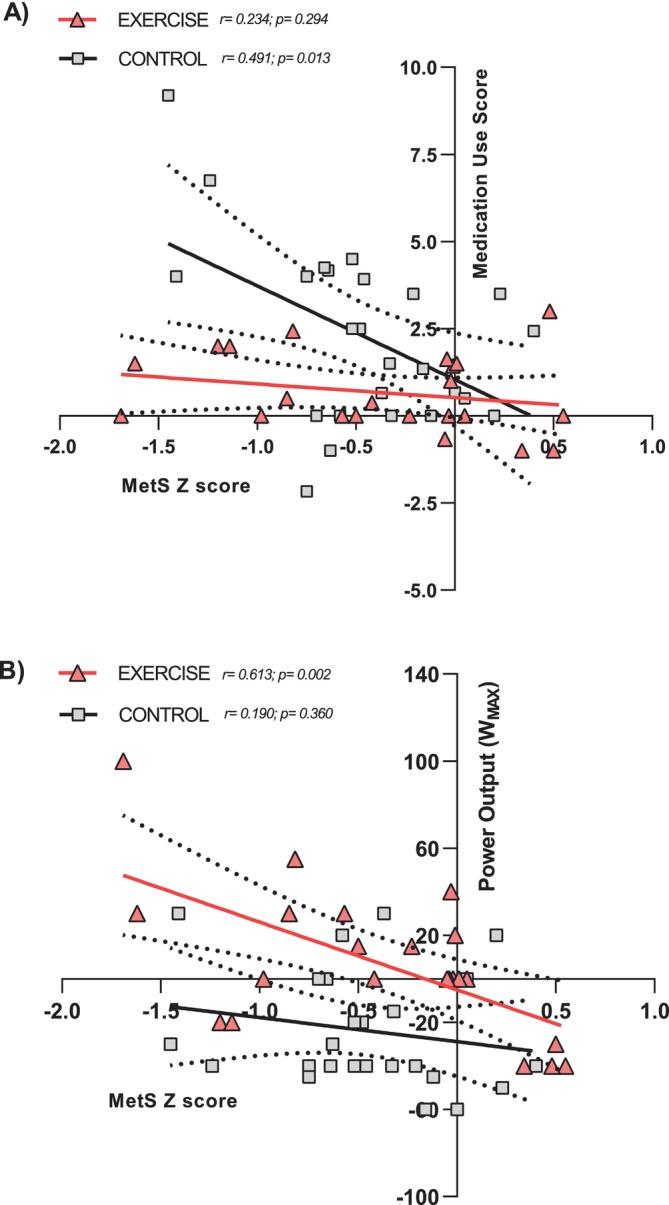
Pearson correlation between improvements in MetS *Z* score (Δ MetS *Z* score) after 8 years of follow‐up and changes in medication use score (Δ medication use score [A]) and W_MAX_ (Δ power output [B]) in the EXERCISE and CONTROL.

Stepwise linear regression model predicting the evolution of MetS *Z* score after 8 years included W_MAX_ and medication use, accounting for 31% of the variance in MetS *Z* score (*r* = 0.581; *p* < 0.001). Pearson correlations showed that changes in MetS *Z* score were significantly associated with changes in medication use score in the CONTROL group (*r* = 0.491; *p* = 0.013; Figure 4A) and with W_MAX_ evolution in the EXERCISE group (*r* = 0.613; *p* = 0.002; Figure 4B).

## 4 Discussion

This study was designed to evaluate if repeated yearly exercise training maintains its health‐promoting effects on the clinical management of MetS as individuals age from 50 to 60 years, a key decade where the incidence of cardiometabolic diseases rises exponentially [2]. The CONTROL group, which received lifestyle advice, demonstrated comparable improvements in their MetS *Z* score and body weight to those in the EXERCISE group. This result could lead to the conclusion that ageing is beneficial for the management of MetS and that yearly exercise is worthless. However, a careful record of the subject's medication revealed that the CONTROL group lowered their MetS *Z* score at the expense of tripling the dose of medication to control their hypertension, hyperglycaemia and dyslipidaemia. We have published prior observations of this apparent paradox [28] but wanted to extend our observations for 8 years to observe if either exercise or medicine prescription reduced their effects with ageing. The main finding is that both healthcare strategies (exercise and polymedication) are effective when used in individuals with MetS in the 50 to 60 years age group.

We developed a medication use score that allowed us to accurately examine the changes in the use of medications for the clinical control of MetS (i.e., lipid–glucose–blood pressure‐lowering drugs). The finding that the CONTROL group had similar improvements in blood pressure and blood lipids as the EXERCISE group (all‐time effect *p* < 0.05; Table 1) could be explained by the significant increase in the medication use score of antihypertensive and lipid‐lowering medications (91% and 233%, respectively; Table 2), mainly represented by an increment in the use of statins, beta‐blockers and diuretics in the CONTROL group (see Table S2). Thus, in the CONTROL group, we found a significant association between the improvements in MetS *Z* score and the increases in medication use score (*r* = 0.491, *p* = 0.013, Figure 4A). Increasing medicine intake raises the possibility of adverse side effects and drug interactions when different medicines are prescribed (polypharmacy; [30]). In addition, increasing the dose of medicine results in drug tolerance and decreases its biological effects [S50]. With long‐term use of orally delivered medicines, there are risks of gastrointestinal discomfort and drug metabolism and excretion could overload liver and kidney function [S51, S52]. Lastly, polypharmacy is tied in with increased economic health costs [4]. In contrast, there was no evidence that the same exercise training dose (4 months of HIIT) repeated over the years, had less of an effect on MetS *Z* score reduction when individuals aged from 52 to 60 years. This is remarkable because training commitment was only 4 months per year and this dose of intense aerobic exercise, without concomitant dieting, had equivalent health effects to tripling oral medication use.

MetS is a comorbidity that is strongly associated with the development of premature frailty and polypharmacy [S53–S54]. It is therefore imperative to consider unintentional weight loss, patients' levels of physical activity and diet composition in ageing populations, as these factors are likely to be significant contributors to the onset of risk factors and frailty [31]. As risk factors accrue, the efficacy of individual medications for each component is often inadequate to achieve effective control, necessitating the administration of multiple drugs (see Table S2). As the number of medicines needed to control risk factors and complications increases, so does the potential for adverse effects—side effects, drug–drug interactions, nonadherence and medication prescription errors. Our results suggest that despite 8 years of ageing (from 50 to 60 years old), there was no change in dietary pattern (total calories and nutrient composition) or physical activity levels between groups (see Table S1). This monitoring supports our primary finding that participating in an annual intensive exercise programme while maintaining diet and physical activity reduces the deterioration of the cardiometabolic systems, delaying polypharmacy and likely preventing premature frailty.

Some studies show that intensive lifestyle interventions combining diet and exercise can result in clinically meaningful weight loss (body weight loss > 6.5% [21, 22, 23]). In these studies, individuals reduce obesity‐related conditions such as diabetes, hypertension and dyslipidaemia to the point that clinicians de‐prescribe some medication. In the U‐TURN study, 74% of participants in the lifestyle group reduced their glucose‐lowering medication after 12 months, primarily due to a 7% loss in body weight [23] in a dose–response relationship with exercise volume [S54]. However, follow‐up monitoring of these individuals reveals that only 37% of the individuals in the lifestyle intervention arm of the DPP study maintained weight loss after 3 years [32], and 27% of individuals in the intensive lifestyle intervention arm of the Look AHEAD maintained weight loss after 8 years [33]. In general, it is estimated that only 20% of overweight individuals maintain weight loss after 1 year of participation in a weight loss programme [34]. Eventually, the almost unavoidable weight rebound requires the reintroduction of medication to manage cardiometabolic disease risk [35]. These findings suggest that intervention programmes involving significant weight changes do not sustain the initial efforts of patients over time [S55]. In contrast, our training programme without dietary manipulation elicited a progressive weight reduction (2% and 5% after 4 and 8 years). Due to the progressive loss of body weight, we avoided weight rebound while maintaining medicine use from significantly increasing during the 8 years that data collection lasted.

A cross‐sectional study shows that high muscular strength and cardiorespiratory fitness levels are independently and inversely associated with MetS prevalence [36]. Muscle strength level represents a robust predictor of mortality as individuals age [12], and muscle power is related to independence in daily living activities in older individuals [S56] reasons why some have regarded muscle power as the best clinical hallmark of ageing. Literature evidences a natural reduction of leg muscle power of 1.1%–1.4% per year in middle‐aged individuals (40–60 years; [37]). Coinciding with those studies, the CONTROL group decreased 4% and 14% leg cycling muscle power in 4 and 8 years, respectively. However, the ageing‐induced decline in leg muscle power disappeared when these middle‐aged MetS patients were involved in a 4‐month yearly cycling exercise training intervention. Moreover, we have found that MetS improvements (i.e., *Z* score) were associated with muscle power gains following training (*r* = 0.613, *p* = 0.002, Figure 4B), which aligns with results from previous studies using cross‐sectional data [36]. Therefore, MetS patients benefit from exercise training in their cardiometabolic systems along with protection from age‐induced muscle power decline.

Physical fitness is considered a strong risk factor for chronic disease that is not routinely assessed in clinical practice, and CRF is one of the best predictors of longevity [38]. CRF inevitably declines with age, but the rate of decline accelerates after age 45 years, influenced by factors such as sex, physical activity and body composition [S57]. Metabolic disease and likely MetS reduce CRF improvements with aerobic training [S58]. Therefore, our finding that CRF increased by 14% (3.4 mL·kg^−1^·min^−1^; 1MET; Table 1) after 8 years of follow‐up with a 4‐month high‐intensity aerobic exercise intervention supports the yearly trainability of individuals with MetS, reverses of the expected declines in CRF with age (see Figure S1, Electronic Supporting Information). The EXERCISE group in 8 years of training, increased by 1 MET their CRF which is associated with an 11% reduction in all‐cause mortality [39]. In addition, many of them entered the 9.0 METs category, which is associated with a 50% reduction in mortality risk [40], these findings are potentially important and public health providers should emphasize improvements in population CRF. Our study is not long enough to analyse all‐cause mortality but according to previous data, EXERCISE treatment may be delaying the incidence of major adverse cardiovascular events.

The present study has limitations and strengths worth mentioning. In this RCT, we studied a relatively small sample (*n* = 47) of individuals with MetS which could bring doubt about the clinical relevance of the findings. Although there are ageing studies measuring health variables in athletes (mostly runners) as they became master athletes, few have a control group, and almost none focused on a sample of individuals with cardiometabolic disease. However, in this population, exercise may have the largest impact on reducing the progression of their cardiometabolic disease as they age. We present a myriad of hard clinical health variables (i.e., blood pressure, body composition, carbohydrate and lipid blood profile, muscle power and cardiovascular fitness). Still, we did not take muscle biopsies to study cell signalling or do more detailed genetic blood analysis to investigate by which mechanisms exercise slows ageing. In turn, the major strength of the study is that we conducted an 8‐year‐long randomized controlled trial, which allows us to assess the sustainability of the health benefits of exercise based on the highest level of evidence. Although lifestyle interventions have been demonstrated to be effective at improving health in the context of experiments, it is crucial to ensure their sustained effects over the years if they are to have a meaningful impact on public health.

In this study, we followed during 8 years a group of individuals with MetS while in a randomized fashion, we allocated them either into a supervised yearly intense exercise‐training programme or remained with their normal activity pattern. Paradoxically, both groups similarly improved their MetS *Z* score (a compound of all cardiometabolic risk factors). A closer analysis of the data revealed that the nonexercising CONTROL group reduced their MetS *Z* score at the expense of tripling their oral medication (Table 2 and Figure 2B). This evidence a worsening of their disease that had to be compensated by increasing pharmacological therapy. On the other hand, the EXERCISE group did not need to increase medication in 8 years to improve their MetS. The factor that better predicted MetS evolution was leg cycling power in the EXERCISE group while it was medicine use score in the CONTROL group (Figure 4A,B). In summary, our data suggest that individuals with cardiometabolic disease, who do not exercise regularly as they age, render the control of their disease to pharmacological treatment alone. Lastly, our data suggest that exercise training has similar clinical power to tripling oral medication to control MetS as individuals age 50 to 60 years.

## Conflicts of Interest

The authors declare no conflicts of interest.

## Supporting information


**Data S1** Supplementary Information.

**Figure S1** Evolution of relative VO_2__MAX_ (A) and W_MAX_ (B) during the 8‐year follow‐up. Data is presented as mean ± SD. † Significant change from baseline within each group. ‡ Significant change from 4 years within each group. # Significant difference between EXERCISE and CONTROL groups at that time point (all *p* < 0.05).

**Table S1** Nutrition and physical activity levels by group. Data are presented as mean ± SD.

**Table S2** Eight‐year evolution of medication type by group. Data are presented as number of subjects taking that drug (%).

## References

[1] G. P. Fadini , G. Ceolotto , E. Pagnin , S. de Kreutzenberg , and A. Avogaro , “At the Crossroads of Longevity and Metabolism: The Metabolic Syndrome and Lifespan Determinant Pathways,” Aging Cell 10 (2011): 10–17. 21040402 10.1111/j.1474-9726.2010.00642.x

[2] G. Hirode and R. J. Wong , “Trends in the Prevalence of Metabolic Syndrome in the United States, 2011–2016,” Journal of the American Medical Association 323 (2020): 2526–2528. 32573660 10.1001/jama.2020.4501 PMC7312413

[3] T. Föhr , A. Hendrix , A. Kankaanpää , et al., “Metabolic Syndrome and Epigenetic Aging: A Twin Study,” International Journal of Obesity 48 (2024): 778–787. 38273034 10.1038/s41366-024-01466-x PMC11129944

[4] S. M. Grundy , “Drug Therapy of the Metabolic Syndrome: Minimizing the Emerging Crisis in Polypharmacy,” Nature Reviews Drug Discovery 5 (2006): 295–309. 16582875 10.1038/nrd2005

[5] M. D. DeBoer , S. L. Filipp , and M. J. Gurka , “Use of a Metabolic Syndrome Severity *Z* Score to Track Risk During Treatment of Prediabetes: An Analysis of the Diabetes Prevention Program,” Diabetes Care 41 (2018): 2421–2430. 30275282 10.2337/dc18-1079 PMC6196828

[6] F. Morales‐Palomo , M. Ramirez‐Jimenez , J. F. Ortega , A. Moreno‐Cabañas , and R. Mora‐Rodriguez , “Exercise Training Adaptations in Metabolic Syndrome Individuals on Chronic Statin Treatment,” Journal of Clinical Endocrinology & Metabolism 105 (2019): e1695–e1704. 10.1210/clinem/dgz304 31875915

[7] A. Moreno‐Cabañas , F. Morales‐Palomo , L. Alvarez‐Jimenez , J. F. Ortega , and R. Mora‐Rodriguez , “Effects of Chronic Metformin Treatment on Training Adaptations in Men and Women With Hyperglycemia: A Prospective Study,” Obesity (Silver Spring) 30 (2022): 1219–1230. 35578807 10.1002/oby.23410 PMC9321693

[8] S. Bajpeyi , M. Pasarica , C. Moro , et al., “Skeletal Muscle Mitochondrial Capacity and Insulin Resistance in Type 2 Diabetes,” Journal of Clinical Endocrinology & Metabolism 96 (2011): 1160–1168. 21307136 10.1210/jc.2010-1621 PMC3070252

[9] S. K. Malin and B. Braun , “Impact of Metformin on Exercise‐Induced Metabolic Adaptations to Lower Type 2 Diabetes Risk,” Exercise and Sport Sciences Reviews 44 (2016): 4–11. 26583801 10.1249/JES.0000000000000070

[10] R. G. Walton , C. M. Dungan , D. E. Long , et al., “Metformin Blunts Muscle Hypertrophy in Response to Progressive Resistance Exercise Training in Older Adults: A Randomized, Double‐Blind, Placebo‐Controlled, Multicenter Trial: The MASTERS Trial,” Aging Cell 18 (2019): e13039. 31557380 10.1111/acel.13039 PMC6826125

[11] M. Ramirez‐Jimenez , F. Morales‐Palomo , J. F. Ortega , and R. Mora‐Rodriguez , “Effects of Intense Aerobic Exercise and/or Antihypertensive Medication in Individuals With Metabolic Syndrome,” Scandinavian Journal of Medicine & Science in Sports 28 (2018): 2042–2051. 29771450 10.1111/sms.13218

[12] L. Andersen Lars , R. López‐Bueno , R. Núñez‐Cortés , L. Cadore Eduardo , A. Polo‐López , and J. Calatayud , “Association of Muscle Strength With All‐Cause Mortality in the Oldest Old: Prospective Cohort Study From 28 Countries,” Journal of Cachexia, Sarcopenia and Muscle 15 (2024;n/a): 2756–2764, 10.1002/jcsm.13619. 39439054 PMC11634500

[13] B. Egan and J. R. Zierath , “Exercise Metabolism and the Molecular Regulation of Skeletal Muscle Adaptation,” Cell Metabolism 17 (2013): 162–184. 23395166 10.1016/j.cmet.2012.12.012

[14] M. J. MacInnis and M. J. Gibala , “Physiological Adaptations to Interval Training and the Role of Exercise Intensity,” Journal of Physiology 595 (2017): 2915–2930. 27748956 10.1113/JP273196 PMC5407969

[15] F. Morales‐Palomo , M. Ramirez‐Jimenez , J. F. Ortega , P. L. Lopez‐Galindo , J. Fernandez‐Martin , and R. Mora‐Rodriguez , “Effects of Repeated Yearly Exposure to Exercise‐Training on Blood Pressure and Metabolic Syndrome Evolution,” Journal of Hypertension 35 (2017): 1992–1999. 28594711 10.1097/HJH.0000000000001430

[16] J. A. Laukkanen , F. Zaccardi , H. Khan , S. Kurl , S. Y. Jae , and R. Rauramaa , “Long‐Term Change in Cardiorespiratory Fitness and All‐Cause Mortality: A Population‐Based Follow‐Up Study,” Mayo Clinic Proceedings 91 (2016): 1183–1188. 27444976 10.1016/j.mayocp.2016.05.014

[17] R. Jurca , M. J. Lamonte , C. E. Barlow , J. B. Kampert , T. S. Church , and S. N. Blair , “Association of Muscular Strength With Incidence of Metabolic Syndrome in Men,” Medicine and Science in Sports and Exercise 37 (2005): 1849–1855. 16286852 10.1249/01.mss.0000175865.17614.74

[18] M. Hassinen , T. A. Lakka , L. Hakola , et al., “Cardiorespiratory Fitness and Metabolic Syndrome in Older Men and Women: The Dose Responses to Exercise Training (DR's EXTRA) Study,” Diabetes Care 33 (2010): 1655–1657. 20413523 10.2337/dc10-0124 PMC2890377

[19] T. A. Lakka , D. E. Laaksonen , H. M. Lakka , et al., “Sedentary Lifestyle, Poor Cardiorespiratory Fitness, and the Metabolic Syndrome,” Medicine and Science in Sports and Exercise 35 (2003): 1279–1286. 12900679 10.1249/01.MSS.0000079076.74931.9A

[20] B. J. Fraser , L. Blizzard , M. J. Buscot , et al., “Muscular Strength Measured Across the Life‐Course and the Metabolic Syndrome,” Nutrition, Metabolism, and Cardiovascular Diseases 32 (2022): 1131–1137. 10.1016/j.numecd.2022.01.018 35197213

[21] W. C. Knowler , E. Barrett‐Connor , S. E. Fowler , et al., “Reduction in the Incidence of Type 2 Diabetes With Lifestyle Intervention or Metformin,” New England Journal of Medicine 346 (2002): 393–403. 11832527 10.1056/NEJMoa012512 PMC1370926

[22] M. A. Espeland , H. A. Glick , A. Bertoni , et al., “Impact of an Intensive Lifestyle Intervention on Use and Cost of Medical Services Among Overweight and Obese Adults With Type 2 Diabetes: The Action for Health in Diabetes,” Diabetes Care 37 (2014): 2548–2556. 25147253 10.2337/dc14-0093 PMC4140155

[23] M. Y. Johansen , C. S. MacDonald , K. B. Hansen , et al., “Effect of an Intensive Lifestyle Intervention on Glycemic Control in Patients With Type 2 Diabetes: A Randomized Clinical Trial,” Journal of the American Medical Association 318 (2017): 637–646. 28810024 10.1001/jama.2017.10169 PMC5817591

[24] C. Höchsmann , J. L. Dorling , C. K. Martin , et al., “Effects of a 2‐Year Primary Care Lifestyle Intervention on Cardiometabolic Risk Factors: A Cluster‐Randomized Trial,” Circulation 143 (2021): 1202–1214. 33557578 10.1161/CIRCULATIONAHA.120.051328 PMC7987882

[25] F. Magkos , M. Yannakoulia , J. L. Chan , and C. S. Mantzoros , “Management of the Metabolic Syndrome and Type 2 Diabetes Through Lifestyle Modification,” Annual Review of Nutrition 29 (2009): 223–256. 10.1146/annurev-nutr-080508-141200 PMC5653262 19400751

[26] F. Morales‐Palomo , A. Moreno‐Cabañas , M. Ramirez‐Jimenez , et al., “Exercise Reduces Medication for Metabolic Syndrome Management: A 5‐Year Follow‐Up Study,” Medicine and Science in Sports and Exercise 53 (2021): 1319–1325. 33433153 10.1249/MSS.0000000000002591

[27] K. G. Alberti , R. H. Eckel , S. M. Grundy , et al., “Harmonizing the Metabolic Syndrome: A Joint Interim Statement of the International Diabetes Federation Task Force on Epidemiology and Prevention; National Heart, Lung, and Blood Institute; American Heart Association; World Heart Federation; International Atherosclerosis Society; and International Association for the Study of Obesity,” Circulation 120 (2009): 1640–1645. 19805654 10.1161/CIRCULATIONAHA.109.192644

[28] F. Morales‐Palomo , A. Moreno‐Cabañas , L. Alvarez‐Jimenez , J. F. Ortega , and R. Mora‐Rodriguez , “Effect of Yearly Exercise on Medication Expense and Benefit–Cost Ratio in Individuals With Metabolic Syndrome: A Randomized Clinical Trial,” Medicine and Science in Sports and Exercise 55 (2023): 158–166. 36171184 10.1249/MSS.0000000000003053

[29] A. Moreno‐Cabañas , J. F. Ortega , F. Morales‐Palomo , et al., “The Use of a Graded Exercise Test May Be Insufficient to Quantify True Changes in V̇o_2max_ Following Exercise Training in Unfit Individuals With Metabolic Syndrome,” Journal of Applied Physiology 2020, no. 129 (1985): 760–767. 10.1152/japplphysiol.00455.2020 32881617

[30] R. W. Hoel , R. M. Giddings Connolly , and P. Y. Takahashi , “Polypharmacy Management in Older Patients,” Mayo Clinic Proceedings 96 (2021): 242–256. 33413822 10.1016/j.mayocp.2020.06.012

[31] N. Buchmann , D. Spira , M. König , I. Demuth , and E. Steinhagen‐Thiessen , “Frailty and the Metabolic Syndrome—Results of the Berlin Aging Study II (BASE‐II),” Journal of Frailty & Aging. 8 (2019): 169–175.31637401 10.14283/jfa.2019.15

[32] R. R. Wing , R. F. Hamman , G. A. Bray , et al., “Achieving Weight and Activity Goals Among Diabetes Prevention Program Lifestyle Participants,” Obesity Research 12 (2004): 1426–1434. 15483207 10.1038/oby.2004.179 PMC2505058

[33] Eight‐Year Weight Losses With An Intensive Lifestyle Intervention: The Look AHEAD Study,” Obesity (Silver Spring) 22 (2014): 5–13. 24307184 10.1002/oby.20662 PMC3904491

[34] R. R. Wing and S. Phelan , “Long‐Term Weight Loss Maintenance,” American Journal of Clinical Nutrition 82 (2005): 222s–225s. 16002825 10.1093/ajcn/82.1.222S

[35] E. W. Flanagan , R. Spann , S. E. Berry , et al., “New Insights in the Mechanisms of Weight‐Loss Maintenance: Summary From a Pennington Symposium,” Obesity (Silver Spring) 31 (2023): 2895–2908. 37845825 10.1002/oby.23905 PMC10915908

[36] R. Jurca , M. J. Lamonte , T. S. Church , et al., “Associations of Muscle Strength and Fitness With Metabolic Syndrome in Men,” Medicine and Science in Sports and Exercise 36 (2004): 1301–1307. 15292736 10.1249/01.mss.0000135780.88930.a9

[37] J. Alcazar , C. Rodriguez‐Lopez , C. Delecluse , M. Thomis , and E. Van Roie , “Ten‐Year Longitudinal Changes in Muscle Power, Force, and Velocity in Young, Middle‐Aged, and Older Adults,” Journal of Cachexia, Sarcopenia and Muscle 14 (2023): 1019–1032. 36788413 10.1002/jcsm.13184 PMC10067493

[38] J. J. Lang , S. A. Prince , K. Merucci , et al., “Cardiorespiratory Fitness Is a Strong and Consistent Predictor of Morbidity and Mortality Among Adults: An Overview of Meta‐Analyses Representing Over 20.9 Million Observations From 199 Unique Cohort Studies,” British Journal of Sports Medicine 58 (2024): 556–566. 38599681 10.1136/bjsports-2023-107849 PMC11103301

[39] J. A. Laukkanen , N. M. Isiozor , and S. K. Kunutsor , “Objectively Assessed Cardiorespiratory Fitness and All‐Cause Mortality Risk: An Updated Meta‐Analysis of 37 Cohort Studies Involving 2,258,029 Participants,” Mayo Clinic Proceedings 97 (2022): 1054–1073. 35562197 10.1016/j.mayocp.2022.02.029

[40] P. Kokkinos , C. Faselis , I. B. H. Samuel , et al., “Cardiorespiratory Fitness and Mortality Risk Across the Spectra of Age, Race, and Sex,” Journal of the American College of Cardiology 80 (2022): 598–609. 35926933 10.1016/j.jacc.2022.05.031

