# Supplementary material for: Long‐Term Effects of High‐Intensity Aerobic Training on Metabolic Syndrome: An 8‐Year Follow‐Up Randomized Clinical Trial
Source: J Cachexia Sarcopenia Muscle. 2025 Apr 2;16(2):e13780. doi: 10.1002/jcsm.13780 (PMC11962644; doi:10.1002/jcsm.13780)
Supplement: Supplementary file 1 — Data S1 Supplementary Information. [file JCSM-16-e13780-s003.docx]

**Supplementary material**

**References**

**S41**. Mikus CR, Boyle LJ, Borengasser SJ, Oberlin DJ, Naples SP, Fletcher J, et al. Simvastatin impairs exercise training adaptations. J Am Coll Cardiol. 2013;62:709-14.

**S42**. Boulé NG, Kenny GP, Larose J, Khandwala F, Kuzik N, Sigal RJ. Does metformin modify the effect on glycaemic control of aerobic exercise, resistance exercise or both? Diabetologia. 2013;56:2378-82.

**S43**. Miller BF, Thyfault JP. Exercise-Pharmacology Interactions: Metformin, Statins, and Healthspan. Physiology (Bethesda). 2020;35:338-47.

**S44**. Tjønna AE, Lee SJ, Rognmo Ø, Stølen TO, Bye A, Haram PM, et al. Aerobic interval training versus continuous moderate exercise as a treatment for the metabolic syndrome: a pilot study. Circulation. 2008;118:346-54.

**S45**. Johnson JL, Slentz CA, Houmard JA, Samsa GP, Duscha BD, Aiken LB, et al. Exercise training amount and intensity effects on metabolic syndrome (from Studies of a Targeted Risk Reduction Intervention through Defined Exercise). Am J Cardiol. 2007;100:1759-66.

**S46**. Craig CL, Marshall AL, Sjostrom M, Bauman AE, Booth ML, Ainsworth BE, et al. International physical activity questionnaire: 12-country reliability and validity. Med Sci Sports Exerc. 2003;35:1381-95.

**S47**. Vilaseca Canals J, Espinàs Boquet J. Guía terapéutica en Atención Primaria; basada en la selección razonada de medicamentos (Therapeutic guide in Primary Care; based on the reasoned selection of drugs). 6th edition ed. Barcelona (Spain): semFYC ediciones; 2016.

**S48**. WHO. WHO Collaborating Centre for Drug Statistics Methodology. Guidelines for ATC classification and DDD assignment 2022. Oslo, Norway: Norwegian Inst Publ Hlth; 2021.

**S49**. Guio de Prada V, Ortega JF, Morales-Palomo F, Ramirez-Jimenez M, Moreno-Cabañas A, Mora-Rodriguez R. Women with metabolic syndrome show similar health benefits from high-intensity interval training than men. PLoS One. 2019;14:e0225893.

**S50**. Holford NHG, Sheiner LB. Understanding the Dose-Effect Relationship. Clinical Pharmacokinetics. 1981;6:429-53.

**S51**. Alhassani RY, Bagadood RM, Balubaid RN, Barno HI, Alahmadi MO, Ayoub NA. Drug Therapies Affecting Renal Function: An Overview. Cureus. 2021;13:e19924.

**S52**. Lammert C, Einarsson S, Saha C, Niklasson A, Bjornsson E, Chalasani N. Relationship between daily dose of oral medications and idiosyncratic drug-induced liver injury: search for signals. Hepatology. 2008;47:2003-9.

**S53**. Hanlon P, Nicholl BI, Jani BD, Lee D, McQueenie R, Mair FS. Frailty and pre-frailty in middle-aged and older adults and its association with multimorbidity and mortality: a prospective analysis of 493 737 UK Biobank participants. The Lancet Public Health. 2018;3:e323-e32.

**S54**. MacDonald CS, Johansen MY, Nielsen SM, Christensen R, Hansen KB, Langberg H, et al. Dose-Response Effects of Exercise on Glucose-Lowering Medications for Type 2 Diabetes: A Secondary Analysis of a Randomized Clinical Trial. Mayo Clin Proc. 2020;95:488-503.

**S55**. Katzmarzyk PT, Mire EF, Horswell R, Chu ST, Zhang D, Martin CK, et al. Four-year follow-up of weight loss maintenance using electronic medical record data: The PROPEL trial. Obesity Science & Practice. 2024;10:e70017.

**S56**. Foldvari M, Clark M, Laviolette LC, Bernstein MA, Kaliton D, Castaneda C, et al. Association of muscle power with functional status in community-dwelling elderly women. J Gerontol A Biol Sci Med Sci. 2000;55:M192-9.

**S57**. Jackson AS, Sui X, Hébert JR, Church TS, Blair SN. Role of lifestyle and aging on the longitudinal change in cardiorespiratory fitness. Arch Intern Med. 2009;169:1781-7.

**S58**. Soares RN, Lessard SJ. Low Response to Aerobic Training in Metabolic Disease: Role of Skeletal Muscle. Exercise and Sport Sciences Reviews. 2024;52:47-53.
