# Supplementary material for: Long‐Term Effects of High‐Intensity Aerobic Training on Metabolic Syndrome: An 8‐Year Follow‐Up Randomized Clinical Trial
Source: J Cachexia Sarcopenia Muscle. 2025 Apr 2;16(2):e13780. doi: 10.1002/jcsm.13780 (PMC11962644; doi:10.1002/jcsm.13780)
Supplement: Supplementary file 2 — Figure S1 Evolution of relative VO2 MAX (A) and WMAX (B) during the 8‐year follow‐up. Data is presented as mean ± SD. † Significant change from baseline within each group. ‡ Significant change from 4 years within each group. # Significant difference between EXERCISE and CONTROL groups at that time point (all p < 0.05). [file JCSM-16-e13780-s002.docx]

**Figure S1, Electronic Supporting Information.** Evolution of relative VO_2MAX_ (A) and W_MAX_ (B) during the 8-year follow-up. Data is presented as mean ± SD. † Significant change from baseline within each group. ‡ Significant change from 4 years within each group. # Significant difference between EXERCISE and CONTROL groups at that time point (all P<0.05).
