# Supplementary material for: Long‐Term Effects of High‐Intensity Aerobic Training on Metabolic Syndrome: An 8‐Year Follow‐Up Randomized Clinical Trial
Source: J Cachexia Sarcopenia Muscle. 2025 Apr 2;16(2):e13780. doi: 10.1002/jcsm.13780 (PMC11962644; doi:10.1002/jcsm.13780)
Supplement: Supplementary file 3 — Table S1 Nutrition and physical activity levels by group. Data are presented as mean ± SD. [file JCSM-16-e13780-s004.docx]

**Table S1. Electronic Supporting Information.** Nutrition and physical activity levels by group. Data are presented as mean ± SD

|  |  | **EXERCISE (n=22)** | | |  | **CONTROL (n=25)** | | |  | **P value (ɳ^2^)** | |
| --- | --- | --- | --- | --- | --- | --- | --- | --- | --- | --- | --- |
|  |  | **Baseline** | **4 years** | **8 years** |  | **Baseline** | **4 years** | **8 years** |  | **Time** | **Time x Group** |
| Total calorie intake (kcal/day) |  | 2144±312 | 1913±400 | 1881±476 |  | 2168±343 | 2071±387 | 2029±400 |  | 0.051 (0.13) | 0.643 (0.02) |
| % carbohydrate |  | 50±8 | 48±7 | 48±7 |  | 47±8 | 47±7 | 46±8 |  | 0.882 (0.01) | 0.768 (0.04) |
| % fat |  | 33±11 | 31±9 | 32±13 |  | 33±11 | 34±7 | 34±10 |  | 0.807 (0.01) | 0.853 (0.01) |
| % protein |  | 18±3 | 19±3 | 19±3 |  | 19±2 | 18±3 | 20±3 |  | **0.002 (0.24)** | 0.796 (0.01) |
| Physical activity (steps/day) |  | 6834±1915 | 7314±1883 | 7189±1892 |  | 7562±1870 | 7991±2097 | 7779±1825 |  | 0.585 (0.03) | 0.980 (0.00) |
| Time standing (min/day) |  | 156±54 | 151±54 | 167±46 |  | 150±49 | 152±54 | 155±54 |  | 0.619 (0.02) | 0.849 (0.01) |
| Time in supine rest (min/day) |  | 467±78 | 493±57 | 476±70 |  | 484±73 | 479±78 | 514±56 |  | 0.459 (0.04) | 0.178 (0.08) |
