# Supplementary material for: Long‐Term Effects of High‐Intensity Aerobic Training on Metabolic Syndrome: An 8‐Year Follow‐Up Randomized Clinical Trial
Source: J Cachexia Sarcopenia Muscle. 2025 Apr 2;16(2):e13780. doi: 10.1002/jcsm.13780 (PMC11962644; doi:10.1002/jcsm.13780)
Supplement: Supplementary file 4 — Table S2 Eight‐year evolution of medication type by group. Data are presented as number of subjects taking that drug (%). [file JCSM-16-e13780-s001.docx]

**Table S2. Electronic Supporting Information****.** Eight-year evolution of medication type by group. Data are presented as number of subjects taking that drug (%).

|  |  | **EXERCISE (n=22)** | | |  |  | **CONTROL (n=25)** | | |  |
| --- | --- | --- | --- | --- | --- | --- | --- | --- | --- | --- |
|  |  | **Baseline** | **4 years** | **8 years** |  |  | **Baseline** | **4 years** | **8 years** |  |
| **Lipid-lowering** |  |  |  |  |  |  |  |  |  |  |
| Statins |  | 8 (36) | 7 (32) | 7 (32) |  |  | 10 (40) | 12 (48) | 16 (64) ^†#^ |  |
| Ezetimibe |  | 0 (0) | 0 (0) | 2 (9) |  |  | 0 (0) | 1 (4) | 2 (8) |  |
| Fibrates |  | 3 (14) | 2 (9) | 1 (5) |  |  | 2 (8) | 2 (8) | 4 (16) |  |
| **Glucose-lowering** |  |  |  |  |  |  |  |  |  |  |
| Biguanides |  | 4 (18) | 6 (27) | 7 (32) |  |  | 3 (12) | 6 (24) | 6 (24) |  |
| DPP-4 |  | 0 (0) | 2 (9) | 1 (5) |  |  | 3 (12) | 4 (16) | 3 (12) |  |
| Sulphonylureas |  | 0 (0) | 0 (0) | 0 (0) |  |  | 1 (4) | 1 (4) | 1 (4) |  |
| SGLT-2 |  | 0 (0) | 1 (5) | 2 (9) |  |  | 0 (0) | 1 (4) | 4 (16) ^†^ |  |
| Meglitinides |  | 0 (0) | 0 (0) | 0 (0) |  |  | 0 (0) | 0 (0) | 1 (4) |  |
| GLP-1 |  | 0 (0) | 0 (0) | 1 (5) |  |  | 0 (0) | 0 (0) | 2 (8) |  |
| **Blood pressure-lowering** |  |  |  |  |  |  |  |  |  |  |
| ACE inhibitors |  | 2 (9) | 3 (14) | 2 (9) |  |  | 3 (12) | 5 (20) | 6 (24) |  |
| ARB |  | 12 (55) | 13 (59) | 14 (64) |  |  | 5 (20) ^#^ | 5 (20) ^#^ | 9 (36) |  |
| Calcium-channel blocker |  | 4 (18) | 3 (14) | 5 (23) |  |  | 3 (12) | 3 (12) | 3 (12) |  |
| Thiazide diuretics |  | 5 (23) | 6 (27) | 8 (36) |  |  | 5 (20) | 7 (28) | 11 (44) ^†#^ |  |
| β-Blockers |  | 0 (0) | 1 (5) | 1 (5) |  |  | 2 (8) | 3 (12) | 6 (24) ^†^ |  |
| Alpha-Blockers |  | 0 (0) | 0 (0) | 1 (5) |  |  | 0 (0) | 1 (4) | 1 (4) |  |

DPP-4, dipeptidyl peptidase-4 inhibitor; SGLT-2, sodium-glucose co-transporter-2; GLP-1, glucagon-like peptide 1; ACEi, angiotensin-converting enzyme inhibioirs; ARB, angiotensin receptor blocker;

Between-group difference using chi-square χ2 Test.

Within-group difference using Cochran Q Test.

^†^ Significant change from baseline within each group.

^‡^ Significant change from 4 years within each group.

^#^ Significant difference between EXERCISE and CONTROL groups at that time point (all P<0.05).
